# Ewing’s sarcoma of proximal femur: case report of extreme osteotomy with 3D-printed prosthesis for the reconstruction

**DOI:** 10.3389/fbioe.2023.1248330

**Published:** 2023-10-09

**Authors:** Xiaoying Niu, Wen Tian, Xiaoxiao Liang, Weitao Yao, Peng Zhang

**Affiliations:** Department of Orthopedic and Soft Tissue, The Affiliated Cancer Hospital of Zhengzhou University & Henan Cancer Hospital, Zhengzhou, China

**Keywords:** Ewing’s sarcoma, proximal femur, 3D-printed, reconstruction, bone tumor

## Abstract

**Background:** Resection and reconstruction of malignant bone tumors at the proximal femur in adolescent patients has remained a clinical challenge. Considering the growth and development requirements of adolescents, there is no unified standard for the reconstruction of bone defects at the proximal femur. Here, we report a case of 3D-printed titanium alloy customized prosthesis for the construction of proximal femoral bone defects in an adolescent patient with Ewing’s sarcoma of the proximal femur.

**Case presentation:** A 7-year-old female patient presented to a local hospital with left hip pain, and was diagnosed with Ewing’s sarcoma on the proximal left femur. The patient received two courses of neoadjuvant chemotherapy before surgery according to the standard protocol. Considering growth and development problems associated with adolescents, we adopted a customized 3D-printed prosthesis of proximal femur for preservation of the femoral head and part of the femoral neck in the affected limb. Clinical outcomes, recorded after 12 months of follow-up, revealed excellent functional recovery and satisfactory functional scores of the affected limb, with no immediate complications.

**Conclusion:** 3D-printed prosthesis is a feasible method for preserving femoral head and reconstruction of bone defects in adolescents’ proximal femur.

## Background

Osteosarcoma and Ewing’s sarcoma are the most frequent primary malignant bone tumors among children (Ottaviani and Jaffe, 2009). The proximal femur is a common site of primary malignant bone tumors, including chondrosarcoma, Ewing’s sarcoma and osteosarcoma (Unni, 1996). Treatments are multidisciplinary, mainly a combination of chemotherapy, radiotherapy and surgery (Cirstoiu et al., 2019). Amputation was previously considered the principal surgical treatment assuring a radical margin. However, improved chemotherapy schedules have not only been shown to spare the limb but also improve patient survival (Ferrari and Palmerini, 2007; Hesla et al., 2021). Currently, limb salvage surgery is considered the standard treatment modality for malignant tumors (Goryń et al., 2019). In cases where sarcoma involves the metaphysis, there is need for the joint to be excised together with the tumor in order to achieve oncologically safe margins. Advancements in imaging technology and chemotherapy, especially the development of magnetic resonance imaging (MRI), have improved identification of tumor boundaries, thereby making it possible for clinicians to efficiently excise the tumor via transmetaphysis osteotomy, and spare the joint thereby resulting in better functional outcomes. To achieve this joint-sparing osteotomy, surgeons need to perform a precise excision. However, it is extremely challenging to reconstruct the defect in cases where the residual host bone is short (Wong and Kumta, 2013; Tsuda et al., 2019). Computed tomography (CT) and MRI data can be fused, with assistance of computer navigation, for identification of tumor boundaries. This enables surgeons to set close but tumor-free surgical margins and excise the tumor more precisely. This technology has been successfully applied in complex sites, such as the spine, sacrum, and pelvis (Wong and Kumta, 2013; Li et al., 2014; Yang et al., 2016; Abraham et al., 2018; Farfalli et al., 2018).

Notably, the lesion is still far from the proximal epiphyseal line and femoral head in some patients. The recent emergence and rapid development of personalized 3D-printed prostheses have enabled preservation of the proximal femoral epiphyseal line and femoral head, and allowed removal of the tumor bone. Notably, a 3D-printed proximal femoral prosthesis can preserve the epiphyseal line, femoral head, joint capsule, round ligament and other structures, thereby improving postoperative rehabilitation of patients and the function of the affected limb.

## Case description

### History

**FIGURE 1:**
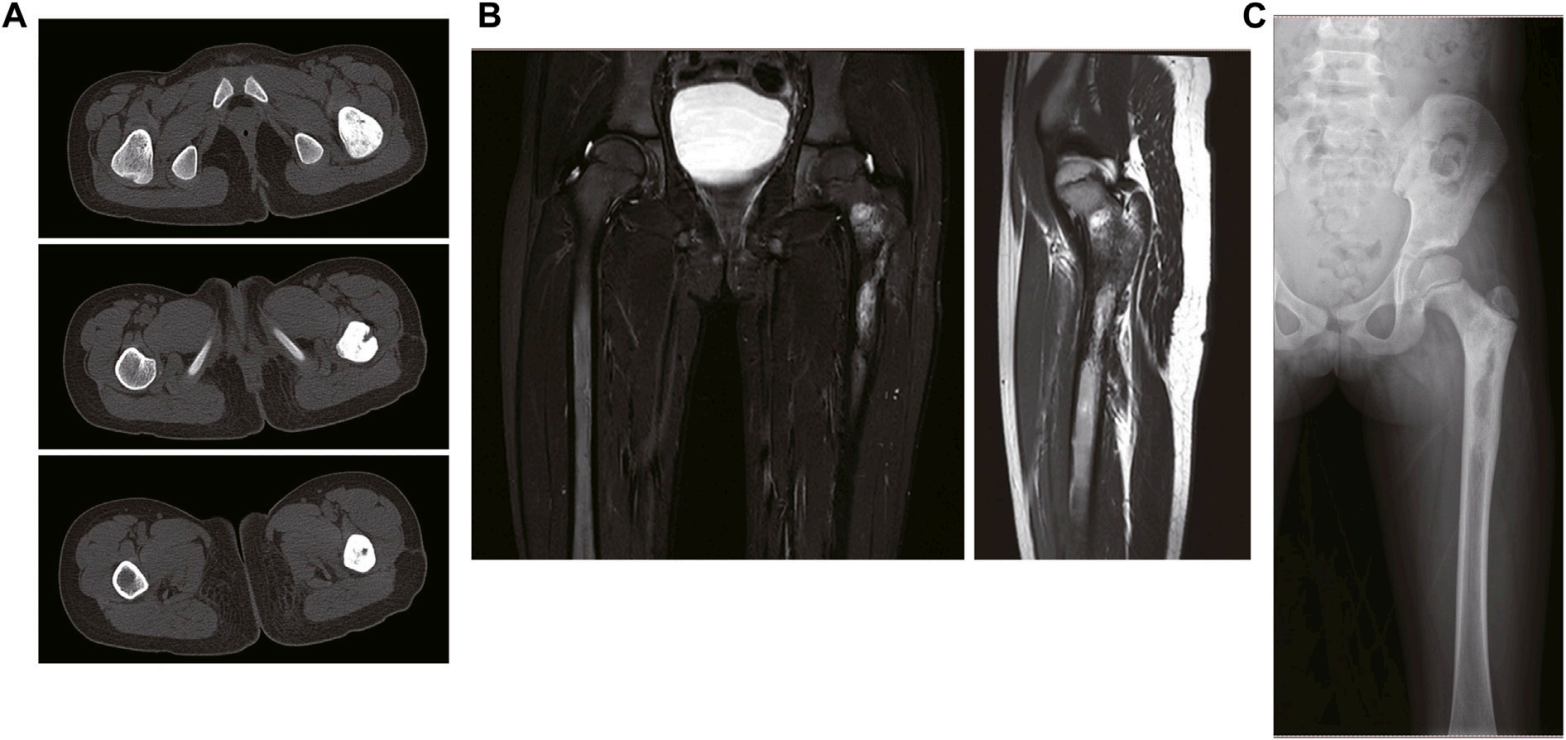
The imaging data of the patient on admission. **(A)** The CT results of the patient. **(B)** The MRI results of the patient. **(C)** The X-ray results of the patient.

A 7-year-old girl was admitted to a local hospital 2 years ago because of pain in her left hip, which was subsequently diagnosed as “osteomyelitis”. Symptomatic anti-inflammatory treatment did confer any significant improvement, while pathological results from open biopsy revealed a small round cell malignant tumor. Expert opinion from a pathological expert at Henan Anti-Cancer Association suggested that the condition was Ewing’s sarcoma. She was referred to a doctor at our hospital. CT results, at admission, revealed irregular and dense local cortical bone of the left femoral neck and proximal femur (Figure 1A). MRI results revealed semi-patchy abnormal signals in the medullary cavity of the left femoral neck and proximal femur, with evidence of a few patchy abnormal signals in the soft tissue of the left lateral thigh (Figure 1B). X-ray results revealed presence of scattered irregular dense masses in the upper part of the left femur (Figure 1C). In July 2021, she was subjected to 2 chemotherapy courses comprising the “VAC/IE” regimen (Vincristine + Adriamycin + Cyclophosphamide/Isocyclophosphamide + Etoposide). Preoperative neoadjuvant chemotherapy regimen: intravenous injection of vincristine 1.5 mg/m^2^+ Doxorubicin 75 mg/m^2^+ cyclophosphamide 1.2 g/m^2^ on the first day of each cycle, intravenous injection of isocyclophosphamide 1.8 g/m^2^+ etoposide 100 mg/m^2^ on the 15th to 19th day per day. 28 days was one full cycle of chemotherapy.

### Surgical procedures

**FIGURE 2:**
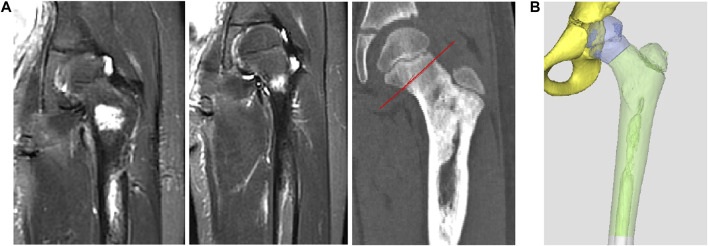
Preoperative osteotomy design planning. **(A)** The determination of osteotomy by MRI. **(B)** The design of preliminary prosthesis by software.

After admission, CT and MRI examinations were performed before neoadjuvant chemotherapy and after chemotherapy, and the imaging data were compared before and after chemotherapy to determine the osteotomy location after excluding the edema area (Figure 2A).

**FIGURE 3:**
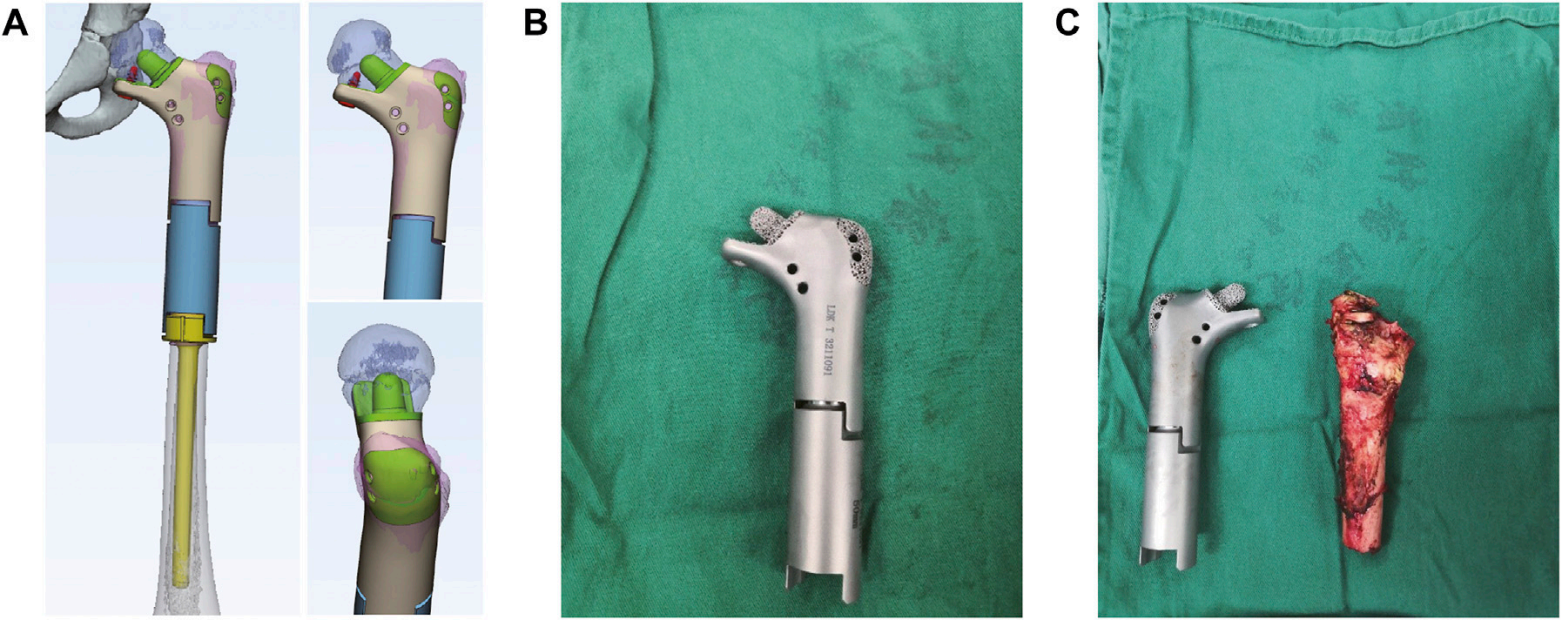
The design of 3D printed prosthesis, the actual prosthesis and the resection of tumor during the operation. **(A)** The design of personal 3D-printed prosthesis. **(B)** The actual prosthesis. **(C)** The resection of tumor during surgery.

Thin-slice CT (0.625 mm thickness) and 3D reconstruction were used as the main data sources, and combined with information from MRI examination. After determining the lesion site and structural details, the original image was reconstructed in 3D and stored in DICOM format. The 3D reconstructed image was obtained by Simics software. Design personalized 3D-printed prostheses for the patient through 3D design software UNIGRAPHICS NX. The porous structure of the prosthesis was designed by MAGICS software (Figure 2B; Figure 3A). The total operation time of the patient was 4 h and 50 min, and the intraoperative blood loss was about 350 mL.

### Prosthesis design

The length of prosthesis was in accordance with the condition of the contralateral side. In order to ensure firm fixation, and owing to the small amount of bone remaining in the proximal femoral neck position, we designed the prosthesis’ proximal medullary needle and proximal contact surface as a horn-like structure, and a reticular pore structure, respectively, in order to facilitate bone ingrowth during later stages. Notably, we designed a locking screw and placed it under the proximal femoral neck in order to prevent rotational shearing force of the prosthesis (Figure 3B; Figure 3C). In addition, a suture pore-like structure was designed at the corresponding positions of the greater and lesser trochanters, then the greater trochanter printed as a 3D grid to facilitate the suture and attachment of tendon insertion.

The operation, performed under general anesthesia, was in the semi-lateral decubitus position with conventional anterolateral approach to the proximal thigh. Briefly, the tumor tissue in the middle and upper femur was exposed, and the proximal femur was osteotomized using an osteotomy guide plate according to the preoperative plan. The direction of the proximal residual femoral neck was then marked for subsequent prosthesis installation. Next, the tumor bone was removed, along the safe boundary around the tumor bone, the 3D printed prosthesis installed and firmly fixed. Finally, two drainage tubes were placed, and the affected limb pressurized with elastic bandages.

**FIGURE 4:**
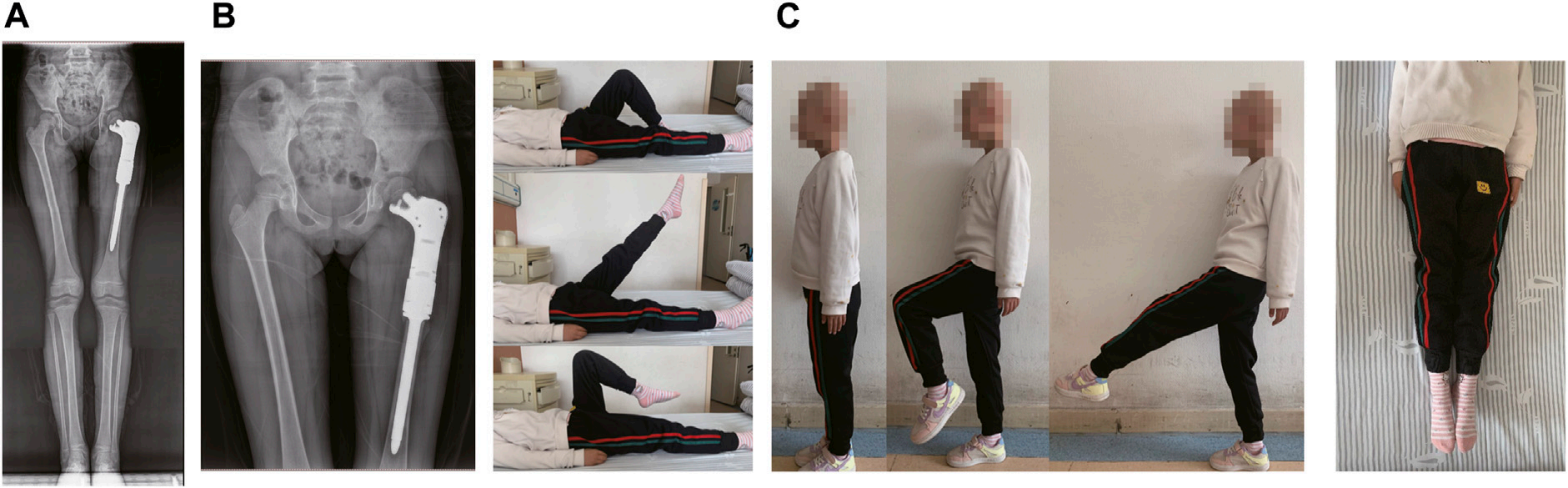
The results of post-operation of the patient. **(A)** Postoperative radiographs at 12 months after the operation. **(B)** The reexamined of functionality of the affected limb at 12 months after surgery. **(C)** The last reexamined of functionality of the affected limb.

Postoperative X-ray film, obtained during follow-up, showed that the prostheses were in good position, and the postoperative recovery was smooth without related complications. Reexamination, performed 12 months after the operation, revealed excellent functionality of the affected limb (Figures 4A, B). And we found no obvious abnormalities observed at 12 months post-operation (Figure 4C).

### Postoperative management

After operation, the affected limb was properly bandaged with elastic bandages under pressure, and intravenous antibiotics were given to prevent postoperative infection. Three to 6 weeks after surgery, the patient walked with partial weight on crutches, and began to gradually remove the crutches at 12–16 weeks and complete weight-bearing activities. And the patient continued the same chemotherapy regimen for 4 weeks after surgery.

## Discussion

The proximal femur is a common site for primary bone tumors, and the most common site of metastatic tumors. Since the introduction of tumor prosthesis in 1980s, extensive resection and reconstruction of the proximal femur have achieved tremendous efficacy in treatment of tumors in the femoral head and neck (Chandrasekar et al., 2009). Studies have shown that reconstruction of segmental bone defects after part resection of the femur may be performed with endoprostheses (Abudu et al., 1996; Ahlmann and Menendez, 2006), distraction osteogenesis (Tsuchiya et al., 1997), allografts (Donati et al., 1993; Cara et al., 1994; Ortiz-Cruz et al., 1997; Muscolo et al., 2004) and autologous grafts (Davidson et al., 2005; Krieg et al., 2007; Puri et al., 2012; Zekry et al., 2019). Notably, custom-made prostheses have the advantage of allowing early mobilization and return to function. Although distraction osteogenesis can provide bone which will develop sufficient biomechanical strength and durability, the procedure is relatively time consuming and demanding, particularly for very long defects and when patients require chemotherapy (Tsuchiya et al., 1997). However, large bone allografts are associated with significant complications, such as development of fractures, non-union and infections. Previous studies have also shown that the rate of non-union in intercalary reconstructions with allografts range from 15% to 71% (Donati et al., 1993; Cara et al., 1994; Ortiz-Cruz et al., 1997; Muscolo et al., 2004), and the there is also a potential risk of transmission of infectious diseases (Tomford, 1995).

Limb salvage surgery must be the goal to pursue when a wide resection of a tumor can be achieved. Improvements in sarcoma treatment therapies has markedly reduced the need for amputation. Notably, the methods for limb reconstruction after resection of malignant bone tumors in children vary depending on the site of resection. Consequently, various types of reconstructions, including massive bone allograft and allograft prosthesis composite have been developed (Groundland et al., 2016). The main problem in children is the small size of bone. In addition, reconstruction of the femur in children is challenging, owing to the fact that surgical resection of tumors in skeletally immature patients is complicated by loss of a physis, with a resultant potential for clinically relevant limb-length discrepancy at the end of the growth (Manfrini et al., 2016; Errani et al., 2019). Inspired by the femoral stem prosthesis and a 3D-printed prosthesis that preserves the epiphysis articular surface of the distal femur, for some malignant bone tumors that do not invade the femoral head, we tried to use 3D customized prosthesis that preserves the femoral head, part of the joint capsule and the femoral neck to build bone defects. Because the patient was young and the condition occurred in a rapidly growing segment of the body, compared with traditional hemiarthroplasty and total femoral prosthesis replacement, this 3D printed customized prosthesis that preserves the femoral head and part of the femoral neck, which is more conducive to the functional recovery of the affected limb after surgery and has less impact on the growth and development of the lower limb.

Reexamination at 16 months follow-up revealed good limb function, and no evidence of complications, which demonstrated feasibility of the osteotomy reconstruction method of proximal femur preserving the epiphysis and femoral head. In addition, the affected limb exhibited good functionality, at both short and medium-term follow-up, with excellent MSTS scores. We found no evidence of loosening, displacement or bone resorption in the prosthesis.

## Conclusion

In conclusion, 3D-printed personalized prosthesis could be a useful treatment for reconstruction of bone defects after extreme osteotomy in Ewing sarcoma patients. The healing of the interface between the 3D printed prosthesis and the residual bone and the good stability of the prosthesis can realize the good functional recovery of the affected limb in the early and middle stages.

## Data Availability

The original contributions presented in the study are included in the article/Supplementary Material, further inquiries can be directed to the corresponding author.
